# MERS-CoV nsp1 impairs the cellular metabolic processes by selectively downregulating mRNAs in a novel granules

**DOI:** 10.1080/21505594.2022.2032928

**Published:** 2022-02-06

**Authors:** Zhaoyi Pan, Yujie Feng, Zhihui Wang, Zhengyang Lei, Qiuju Han, Jian Zhang

**Affiliations:** Institute of Immunopharmaceutical Sciences, School of Pharmaceutical Sciences, Shandong University, Jinan, China

**Keywords:** MERS-CoV, LLPS, ribosome, oxidative phosphorylation, nsp1

## Abstract

MERS-CoV infection can damage the cellular metabolic processes, but the underlying mechanisms are largely unknown. Through screening, we found non-structural protein 1 (nsp1) of MERS-CoV could inhibit cell viability, cell cycle, and cell migration through its endonuclease activity. Transcriptome sequencing revealed that MERS-CoV nsp1 specifically downregulated the mRNAs of ribosomal protein genes, oxidative phosphorylation protein genes, and antigen presentation genes, but upregulated the mRNAs of transcriptional regulatory genes. Further analysis shown nsp1 existed in a novel ribonucleosome complex formed via liquid-liquid phase separation, which did not co-localize with mitochondria, lysosomes, P-bodies, or stress granules. Interestingly, the nsp1-located granules specifically contained mRNAs of ribosomal protein genes and oxidative phosphorylation genes, which may explain why MERS-CoV nsp1 selectively degraded these mRNAs in cells. Finally, MERS-CoV nsp1 transgenic mice showed significant loss of body weight and an increased sensitivity to poly(I:C)-induced inflammatory death. These findings demonstrate a new mechanism by which MERS-CoV impairs cell viability, which serves as a potential novel target for preventing MERS-CoV infection-induced pathological damage.

**Abbreviations:** (Middle East respiratory syndrome coronavirus (MERS-CoV), Actinomycin D (Act D), liquid-liquid phase separation (LLPS), stress granules (SGs), Mass spectrometry (IP-MS), RNA Binding Protein Immunoprecipitation (RIP))

## Introduction

Middle East respiratory syndrome coronavirus (MERS-CoV) broke out in Saudi Arabia in 2012, and was the second coronavirus to seriously affect human health following the outbreak of severe acute respiratory syndrome coronavirus (SARS-CoV) in 2002 [1]. The recent outbreak of SARS-CoV-2 has once again posed a major threat to human health worldwide. In addition to symptoms such as fever, headache, fatigue, and chills, these three viruses can cause severe lung and renal failure, which poses a serious threat to human health [2–4]. Several studies have demonstrated that immune system disorders and oxidative stress caused by viral infection can cause lung and renal failure [5–7]. Moreover, an increasing number of studies have shown that mitochondrial damage and abnormal energy metabolism can promote renal failure and lung damage [8–12]. Mitochondrial injury triggers the production of damage-associated molecular patterns (DAMPs), resulting in activation of the immune system and tissue damage [13]. However, whether SARS-CoV, MERS-CoV, and SARS-CoV-2 can induce organ failure via mitochondrial damage remains unclear.

SARS-CoV, MERS-CoV, and SARS-CoV-2 belong to the β-coronavirus family [14], and harbor a positive-sense, single-stranded RNA of about 30 knt. Two-thirds of the 5’ end of the genome encodes a polyprotein (orf1a and orf1ab), which is then cleaved into 15–16 non-structural proteins (nsps) by papain-like protease nsp3 and 3 C-like protease nsp5. The remaining 3’ end genome encodes four structural proteins: E, N, S, M, and some accessory proteins. Only alpha and beta coronaviruses contain nsp1, which is an important viral virulence protein that acts as an endonuclease to degrade mRNA and inhibit protein translation [15–18]. SARS-CoV nsp1 only specifically cleaves mRNA transcribed by RNA polymerase II [19]. MERS-CoV nsp1 has been reported to inhibit host gene expression by selectively targeting mRNAs transcribed in the nucleus while sparing mRNAs of cytoplasmic origin, such as viral RNA [16]. SARS-CoV and SARS-CoV-2 nsp1 suppress protein translation by interacting with the 40S ribosomal subunit. However, MERS-CoV nsp1 did not bind stably to the 40S subunit [16,20,21], so the mechanism by which MERS-CoV nsp1 inhibits protein translation remains unclear.

Previous studies have found that mRNAs in cells are generally combined with RNA-binding proteins to form membrane-less organelles, also known as cytosolic biomolecular condensates, such as P bodies and stress particles [22]. These organelles usually exhibit regulatory effects on post-transcriptional regulation and translation. Each cytosolic biomolecular condensate contains particular RNA-binding proteins, benefiting RNA transport and localization, supporting catalytic processes, storage and inheritance of specific molecules, buffering noise, and responding to stress [23]. In addition to RNA-binding proteins in cells, viral proteins also form ribonucleoprotein complexes via liquid-liquid phase separation. The most widely studied is the viral nucleocapsid protein, which has an RNA-binding domain, such as SARS-CoV-2 NP [24–27]. However, studies on other viral proteins through liquid-liquid phase separation and cytosolic biomolecular condensate formation are limited. In the present study, we aimed to investigate the relationship between nsp1 and MERS-CoV virulence.

## Results

### MERS-CoV nsp1 inhibits cell viability through its endonuclease activity

**Figure 1.**
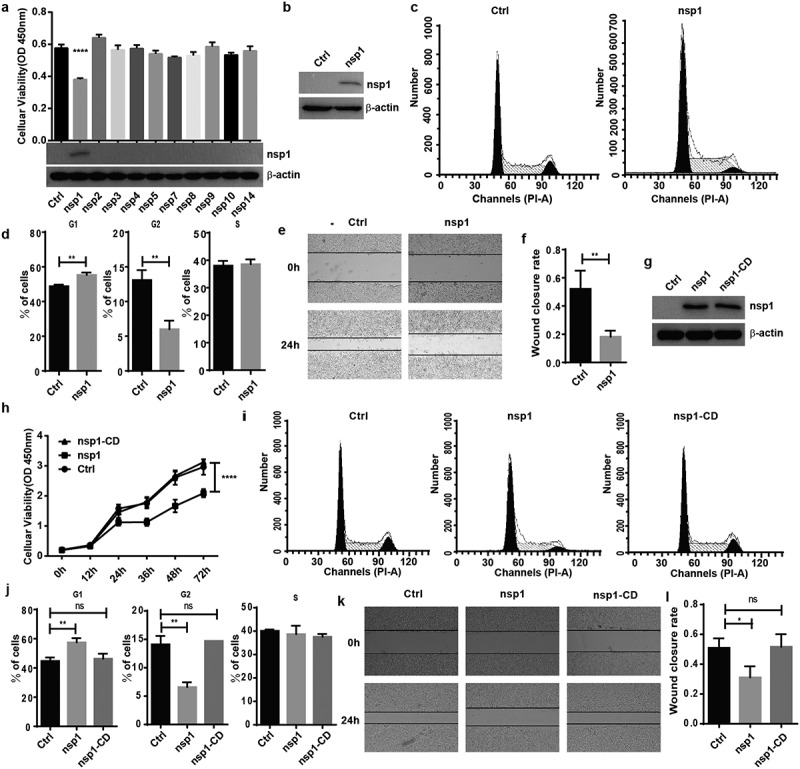
MERS-CoV nsp1 inhibits cell viability through its endonuclease activity. (a) HEK293T cells were transfected with MERS-CoV nsp overexpression vectors, and a CCK8 assay was performed 48 h after transfection. (b) The expression of nsp1 and β-actin was determined by Western blot in HEK293T cells. (c-d) Analysis of the cell cycle in HEK293T cells. (c) Flow cytometry analysis of the cell cycle in HEK293T cells transfected with nsp1 for 36 h. (d) Quantification of flow cytometry cell cycle analysis of nsp1. (e) The effects of MERS-CoV nsp1 on wound healing in HEK293T cells. (f) Quantification of wound closure rate. (g-l) HEK293T cells were transfected with MERS-CoV nsp1, nsp1-CD, or empty vector. (g) The expression of nsp1/nsp1-CD and β-actin was determined by Western blot in HEK293T cells. (h) A CCK8 assay was performed at the indicated time points. (i) Flow cytometry analysis of the cell cycle in HEK293T cells 36 h post-transfection. (j) Quantification of flow cytometry cell cycle analysis of nsp1 and nsp1-CD, at 36 h post ORF introduction. (k) Wound healing of HEK293T cells. (l) Quantification of wound closure rate. Data are mean ± s.d. from at least three independent experiments. *p < 0.05, **p < 0.01, ****p < 0.001.

To test whether MERS-CoV influences host cell viability, overexpression vectors of MERS-CoV nsp encoded by orf1a and orf1ab were cloned and transfected into HEK293T cells. We found that nsp1 significantly inhibited cell viability and growth capacity compared to the other nsps (Figure 1(a)). Furthermore, MERS-CoV nsp1 could inhibit the viability of HEK293T and A549 cells in a dose dependent manner (Fig. S1a and S1b). Cell viability is associated with cell cycle and cell migration ability. Thus, we first confirmed nsp1 expression in HEK293T cells by immunoblot analysis (Figure 1(b)). Flow cytometry analysis indicated that overexpression of nsp1 significantly blocked the cell cycle in the G1 phase (Figures 1(c) and (d)), while the wound healing test showed that nsp1 also disturbed cell migration capacity (Figures 1(e) and (f)). The G1 phase is known as the pre-synthesis phase, and RNA and ribosomes are largely synthesized during this period. A recent study has indicated that MERS-CoV nsp1, as an endonuclease, can cut intracellular mRNAs transcribed in the nucleus, and 146 R and 147 K are responsible for endonuclease activity [16]. To confirm whether the influence of MERS-CoV nsp1 on HEK293T cells was dependent on its endonuclease site, we constructed an nsp1 mutant (nsp1-CD) overexpression vector with mutations at R146A and K147A, and found that nsp1-CD could be expressed well in HEK293T cells (Figure 1(g)). Further experiment indicated nsp1-CD had no significant effect on cell viability (Figure 1(h)), cell cycle (Figure 1(i) and (j)), and wound healing progression (Figures 1(k) and (l)). Similar results were shown in A549 cells that is susceptible to MERS-CoV infection (Fig. S1c-1 f). These results suggest that MERS-CoV nsp1 impacts host cell viability depending on its endonuclease activity.

### MERS-CoV nsp1 selectively downregulates the mRNAs of ribosomal protein genes and oxidative phosphorylation protein genes

**Figure 2.**
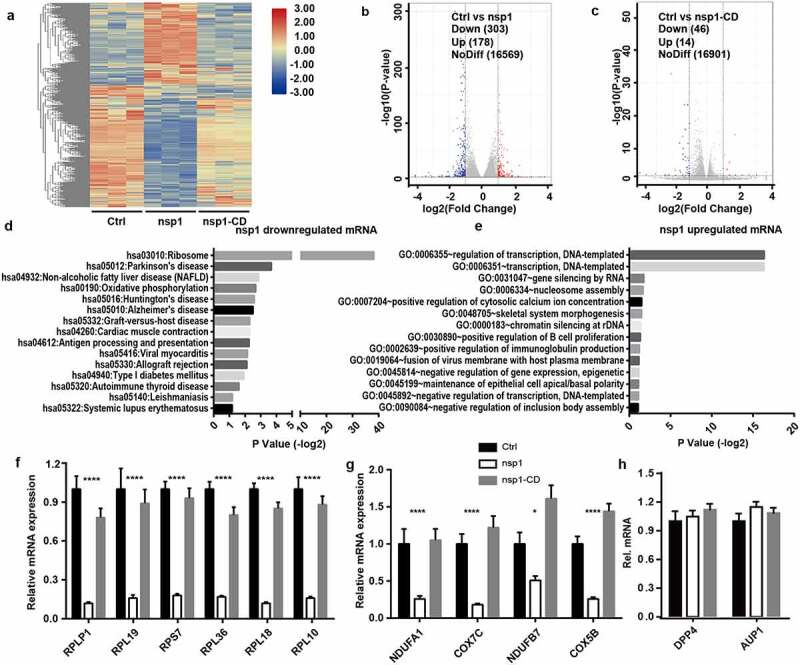
MERS-CoV nsp1 selectively downregulates the mRNAs of ribosomal protein genes and oxidative phosphorylation protein genes. (a) Heatmap depicting the levels of genes influenced by control, nsp1, and nsp1-CD. MERS-CoV nsp1, nsp1-CD, or empty vector were transfected into HEK293T cells and harvested 24 h later. RNA samples were collected for RNA sequencing (n = 3 each group). (b and c) Volcano plot of differentially expressed mRNAs of nsp1 vs. control cells and nsp1-CD vs. vector control cells. (d and e) KEGG enrichment of mRNAs downregulated and upregulated by nsp1. (f and g) Real-time PCR quantification of mRNAs of ribosomal protein genes and mitochondrial protein genes in HEK293T cells transfected with control vector, nsp1, and nsp1-CD. (h) Real-time PCR quantification of mRNAs of *DPP4* and *AUP1*. Data are mean ± s.d. from 3 independent experiments. *p < 0.05, **p < 0.01, ***p < 0.001, ****p < 0.001.

As an endonuclease, coronavirus nsp1 selectively downregulates RNA in host cells. To further confirm whether MERS-CoV nsp1 selectively downregulates nuclear transcribed mRNAs, we investigated the influence of nsp1 and nsp1-CD on the mRNA profile of cells by transcriptome sequencing (Figure 2(a) and Table S1). From the results of the volcano plot, compared to the control group, we found that nsp1 induced thev downregulation of 303 transcripts and upregulation of 178 transcripts by more than two-fold (Figure 2(b)). However, nsp1-CD only induced the downregulation of 46 transcripts and upregulation of 14 transcripts by more than two-fold (FDR adjusted P value < 0.05) (Figure 2(c)). DAVID KEGG enrichment analysis confirmed that the genes downregulated by MERS-CoV nsp1 were mainly rich in ribosomes, oxidative phosphorylation, and antigen presentation (Figure 2(d)), while the upregulated mRNAs were mainly enriched in transcriptional regulation (Figure 2(e)). Furthermore, qPCR was performed to confirm the effects of nsp1 and nsp1-CD on ribosomes and the oxidative phosphorylation of mRNAs. Consistent with the results of transcriptome sequencing, MERS-CoV nsp1 significantly repressed the ribosomal protein genes and oxidative phosphorylation protein genes, while nsp1-CD showed no significant effect on these mRNA levels (Figures 2(f) and (g)). In addition, the mRNAs from other pathways such as MERS-CoV receptor *DPP4* and lipid metabolism related gene *AUP1* were not changed by MERS-CoV nsp1 significantly (Figure 2(h)). These results suggest that MERS-CoV nsp1 affects host cell viability by selectively downregulating the mRNAs of ribosomal protein genes and oxidative phosphorylation protein genes.

### MERS-CoV nsp1 decreases ribosome number and disturbs mitochondrial function

**Figure 3.**
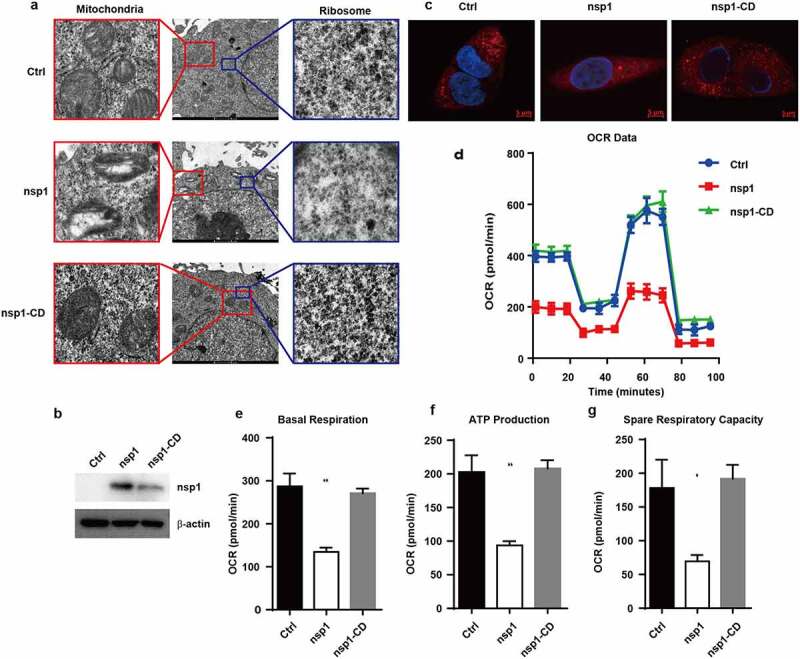
MERS-CoV nsp1 decreases ribosome number and disturbs mitochondrial function. (a) Ribosomes (right) and mitochondria (left) in HEK293T cells transfected with control vector, MERS-CoV nsp1, or nsp1-CD were assessed by electron microscopy. (b) The expression of nsp1 and β-actin in A549 cells was determined by Western blot. (c) Confocal microscopy of Mito-Tracker-Red in A549 cells transfected with control vector, MERS-CoV nsp1, and nsp1-CD. Nuclei were stained with DAPI. The images are representatives of at least three independent experiments. (d) Oxygen consumption rates (OCR) were measured in control vector-, MERS-CoV nsp1-, nsp1-CD-transfected A549 cells treated with oligomycin (1.5 μM), FCCP (1 μM), Rot & AA (0.5 μM). (e-g) Quantification of OCR-based basal respiration, ATP production, and spare respiration capacity analysis of control vector, nsp1, and nsp1-CD- transfected A549 cells. Data are mean ± s.d. from at three independent experiments. *p < 0.05, **p < 0.01.

Ribosomes are organelles for protein synthesis in cells, and oxidative phosphorylation is an important way to produce ATP in cells. Therefore, we subsequently determined whether MERS-CoV nsp1-mediated mRNA downregulation could impair the formation and function of ribosomes and mitochondria. Transmission electron microscopy revealed that MERS-CoV nsp1 decreased the number of ribosomes in the cells, whereas nsp1-CD did not show a significant effect (Figure 3(a)). Meanwhile, nsp1 but not nsp1-CD caused mitochondrial damage, showing reduced mitochondrial cristae and mitochondrial vacuolation (Figure 3(a)). To further confirm whether MERS-CoV nsp1/nsp1-CD disturbs mitochondrial function. Thus, we first confirmed their expression in A549 cells by immunoblot analysis (Figure 3(b)).Mito-Tracker-Red staining further show nsp1 caused mitochondrial damage, whereas nsp1-CD did not significantly influence mitochondria in A549 cells (Figure 3(c)). In addition, nsp1 reduced the membrane potential of mitochondria, which was dependent on its endonuclease site (Fig. S2a). The seahorse test showed that nsp1 significantly suppressed the basal respiration value, ATP production, and space respiratory capacity of A549 cells (Figure 3(d-g)). Similar phenomena were observed in HEK293T cells (Fig. S2b-e). These data indicate that MERS-CoV nsp1 can reduce the number of ribosomes and damage mitochondrial function.

### MERS-CoV nsp1 down-regulates mRNAs by its endonuclease activity

**Figure 4.**
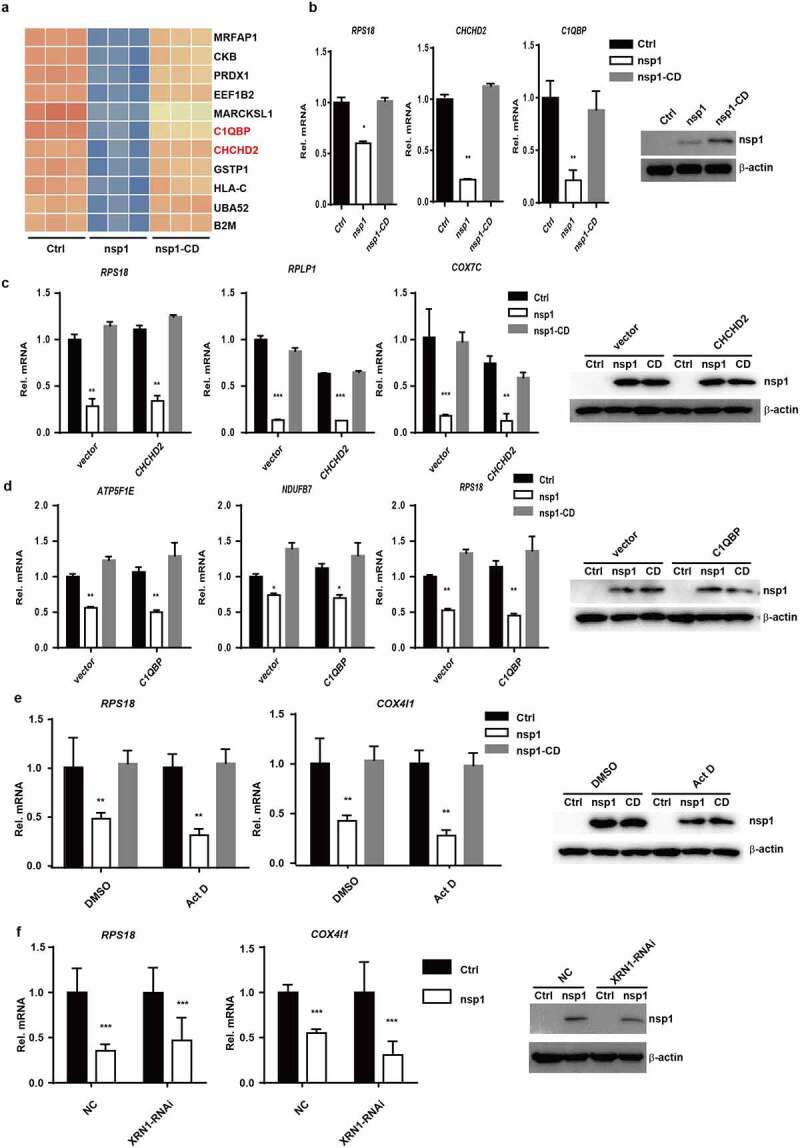
C1QBP can partially alleviate the inhibitory effect of nsp1 on GFP expression. (a) Heatmap depicting the levels of genes influenced by control vector, nsp1, and nsp1-CD (excluding ribosomal protein genes and mitochondrial protein genes). (b) Real-time PCR quantification of RPS18, CHCHD2, and C1QBP in HEK293T cells transfected by control vector, nsp1, and nsp1-CD. (c-d) Effects of CHCHD2 and C1QBP overexpression on recovering the expression of MERS-CoV nsp1-downregulated ribosomal and mitochondrial protein genes. HEK293T cells were co-transfected with control vector, nsp1, or nsp1-CD and CHCHD2 or C1QBP overexpression vectors respectively. Cells were collected after 36 h, and assayed by real-time PCR quantification. (e) Vector, nsp1, or nsp1-CD was transfected into HEK293T cells. 8 h later, these cells were incubated in the absence or presence of ActD. Intracellular RNAs were extracted at 36 h post transfection. (f) Vector or nsp1 was transfected into control or XRN1 knockdown HEK293T cells, the mRNA levels of RPS18 and COX4I1 were analyzed at 36 h post transfection. The expression of nsp1 and β-actin was determined by Western blot. The images are representatives of at least three independent experiments. Data are mean ± s.d. from 3 independent experiments. **p < 0.01, ***p < 0.001.

MERS-CoV nsp1-mediated the downregulation of mRNAs might occur in three ways. One is that MERS-CoV nsp1 induced mRNA degradation through certain key molecules that are responsible for the transcription of ribosomal and mitochondrial protein genes. Previous studies have reported that C1QBP-knockout can cause severe damage to mitochondrial protein synthesis, resulting in serious dysfunction of the mitochondrial respiratory chain [28]. CHCHD2 is an important transcription factor that regulates the transcription of cytochrome C oxidase *COX4* in the mitochondrial respiratory chain [29]. As shown in Figure 4(a) and (b), MERS-CoV nsp1 decreased the mRNA levels of C1QBP and CHCHD2. However, overexpression of CHCHD2 and C1QBP did not rescue the inhibitory effect of nsp1 on the mRNAs (Figure 4(c) and (d)) of ribosomal protein genes and oxidative phosphorylation genes. These results indicate that nsp1-mediated downregulation of the mRNAs of ribosomal protein genes and oxidative phosphorylation protein genes may not be mediated by molecules such as C1QBP and CHCHD2, which are responsible for the transcription of ribosomal and mitochondrial protein genes.

To clarify whether nsp1 down-regulated the mRNA through its endonuclease activity but not through affecting the expression of some important genes such as transcription factors or yet unknown factors, nsp1-overexpressed cells were simultaneously treated with Actinomycin D to inhibit mRNA transcription. The results showed nsp1 could still downregulated the mRNAs of ribosomal protein genes and oxidative phosphorylation protein genes in the presence of Actinomycin D (Figure 4(e)). Finally, Many viruses downregulate host mRNA by triggering an RNA cleavage through host exonuclease XRN1 [19]. However, as shown in Figure 4(f), MERS-CoV nsp1 could still downregulate the mRNAs of ribosomal protein genes and oxidative phosphorylation protein genes even if the XRN1 expression was knockdown, indicating nsp1-mediated mRNAs downregulation is independent of XRN1.Therefore, all these results indicate that MERS-CoV nsp1 selectively down-regulates the mRNAs by its endonuclease activity.

### MERS-CoV nsp1 locates in a novel granules that are rich in the mRNAs nsp1 downregulates

**Figure 5.**
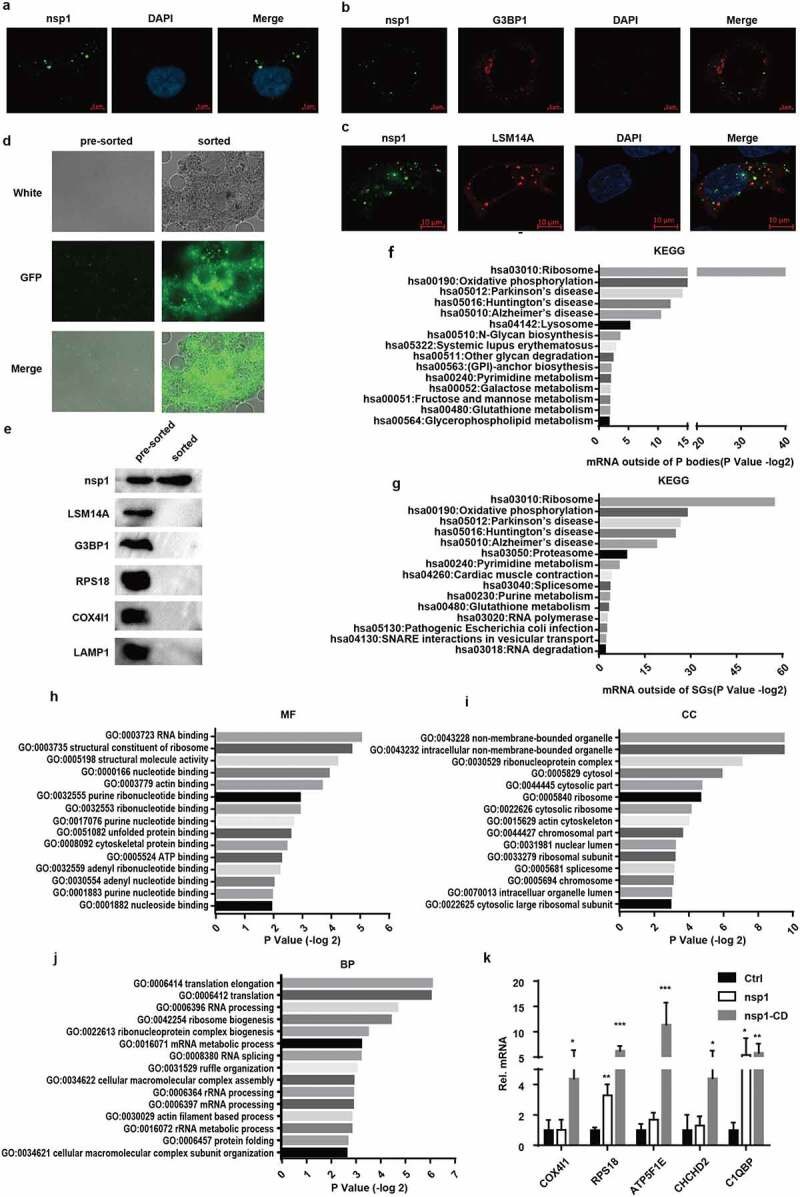
MERS-CoV nsp1 locates in a novel granules rich in the mRNAs of oxidative phosphorylation protein genes and ribosomal protein genes. (a) Confocal microscopy of the cellular localization of MERS-CoV nsp1. HEK293T cells were transfected with pcDNA3.1-nsp1, nsp1 antibody was used to detect the nsp1 expression. (b-c) Confocal microscopy was used to visualize the cellular localization of MERS-CoV nsp1. HEK293T cells were co-transfected with nsp1-GFP and (b) G3BP1-mCherry or (c) LSM14A-mCherry. 100 mM arsenite was used to trigger SGs formation. Nuclei were stained with DAPI. (d and e) Purification of nsp1-GFP granules by anti-GFP-beads. (d) Microscopy of nsp1-GFP granules in pre-sorted and sorted granules. (e) ImMunoblotting of nsp1-GFP granules in pre-sorted and sorted granules. (f) KEGG enrichment of P-body depleted mRNAs. (g) KEGG enrichment of stress granule-depleted mRNAs. (h) Molecular function, (i) biological process, and (j) cellular component terms of nsp1-located granule-enriched proteins. (k) RIP-qPCR analysis of COX4I1, RPS18, ATP5F1E, CHCHD2, and C1QBP. Representative figures of two independent replicates are shown. Data are mean ± s.d. from 3 independent experiments. *p < 0.05, **p < 0.01, ***p < 0.001.

To clarify the mechanism of MERS-CoV nsp1 in selectively downregulating the mRNAs of ribosomal protein genes and oxidative phosphorylation genes, we analyzed the subcellular localization of MERS-CoV nsp1. We observed MERS-CoV nsp1 formed granules that were distributed in the cell (Figure 5(a)), suggesting that nsp1 might cleave the mRNAs only within the granules. Previous studies have shown that mRNA in the cytoplasm forms RNA-protein complexes, such as P bodies and stress granules through liquid-liquid phase separation (LLPS) [30,31]. However, MERS-CoV nsp1 did not co-localize with P bodies, SGs, mitochondria, or lysosomes (Figure 5(b) and (c), Fig. S3a and b). We used agarose beads with a GFP antibody to purify nsp1-located granules in HEK293T cells (Figure 5(d)). The sorted nsp1 granules contained less P bodies, stress particles, lysosomes, mitochondria, and ribosomes (Figure 5(e)), indicating that the granules in which nsp1 was located were different from the known cellular components. To confirm this hypothesis, based on previously published transcriptomes of the P bodies and SGs [32,33], we defined the P body- and SG-depleted mRNAs as the mRNAs enriched in some other ribonucleoprotein complexes. DAVID KEGG enrichment analysis showed that P body- and SG-depleted mRNAs were in line with what we observed MERS-CoV nsp1-downregulated mRNAs, including ribosome protein and oxidative phosphorylation protein genes, and were rich in the pre-sorted fraction (Figure 5(f) and (g)). Only 3 of 43 mRNAs of ribosomal protein genes degraded by nsp1 were not present in the P body- and SG-depleted mRNAs (Fig. S3c), the 10 mRNAs of oxidative phosphorylated proteins degraded by nsp1 were all present (Fig. S3d). These results indicate that nsp1 might exist in an unknown ribonucleoprotein complex that is enriched in mRNAs downregulated by nsp1of ribosomal protein genes and oxidative phosphorylation protein genes, resulting in selective degradation of these mRNAs.

To confirm this speculation that MERS-CoV nsp1 was located in an unknown granule, Mass spectrometry (IP-MS) was conducted to characterize the proteome of nsp1-located granules enriched with the nsp1 antibody. A total of 496 proteins were identified, and 84 proteins were enriched specifically by the nsp1 antibody but not IgG (Fig. S3e, Table S2). Gene ontology (GO) category enrichment characterized nsp1-located granules containing many RNA-binding proteins (Figure 5(h)). Similar to the P bodies and SGs, the nsp1-located granules were non-membrane-bound organelles consisting of ribonucleoprotein complexes (Figure 5(i)). The biological processes of nsp1 specifically interacting proteins were translation, ribonucleoprotein biogenesis, RNA processing, and RNA metabolic processes (Figure 5(j)), indicating that the nsp1-located granules were ribonucleoproteins rich in RNAs.

In order to identify whether the nsp1-located granules were rich in mRNAs of ribosome protein genes and oxidative phosphorylation protein genes, RNA binding protein immunoprecipitation (RIP) was performed using the nsp1 antibody, followed by qPCR analysis. Compared to the control, nsp1-located granules and nsp1-CD-located granules were rich in the mRNAs of ribosomal protein genes and oxidative phosphorylation protein genes, whereas the mRNAs of nsp1 and apoptosis-related genes were almost undetectable and showed no significant difference among the control, nsp1, and nsp1-CD (Figure 5(k) and S3f). Notably, the mRNA levels of ribosomal protein genes and oxidative phosphorylation protein genes in the nsp1 granules were lower than those in the nsp1-CD granules. These results indicate that nsp1-located granules are novel ribonucleoprotein complexes that are specifically rich in the mRNAs of ribosomal protein genes and oxidative phosphorylation protein genes, thereby selectively downregulating the mRNAs of these genes in the granules.

### MERS-CoV nsp1 transgenic mice are more sensitive to poly(I:C)-induced death

**Figure 6.**
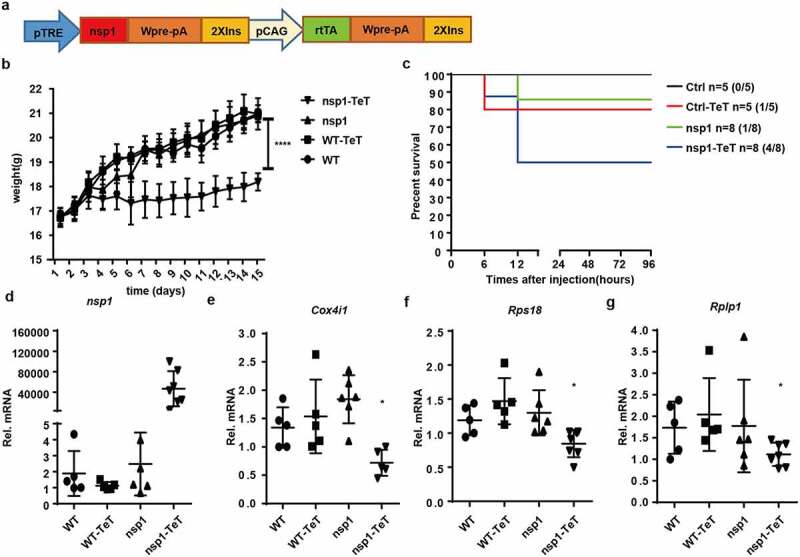
MERS-CoV nsp1 transgenic mice are more sensitive to poly(I:C)-induced death. (a) Design strategy of tetracycline-induced nsp1 transgenic mice. (b) WT and nsp1 transgenic mice of 4–5 weeks were fed with tetracycline or not, and the body weight was recorded once a day for 2 weeks. (c) Sex and age matched WT and nsp1 transgenic mice were fed tetracycline for 2 weeks and then injected intraperitoneally with poly(I:C) plus D-galactosamine. The survival of these mice was monitored every 12 h for 4 days. (d-g) Real-time PCR quantification of nsp1, Cox4i1, Rps18, and Rplp1 mRNAs in the lung tissues of WT and nsp1 mice. Data are representatives or mean± s.d.; n = 5 mice per group. *p < 0.05, **p < 0.01, ***p < 0.001, ****p < 0.001.

To study the effect of MERS-CoV nsp1 in vivo, we constructed a tetracycline-induced nsp1-expression transgenic mouse model (Figure 6(a)). There was no significant difference in morphology, behavior, or growth between nsp1 transgenic mice and wild-type (WT) mice. However, after treatment with tetracycline, the growth of nsp1 transgenic mice was slower than that of mice in the other groups (Figure 6(b)) and was more sensitive to poly(I:C)/ D-galactosamine-induced death (Figure 6(c)). Further analysis demonstrated that nsp1 was significantly induced in the lung tissues of nsp1 transgenic mice (Figure 6(d)), which was accompanied by a decrease in the oxidative phosphorylation protein gene *Cox4i1* mRNA and ribosomal protein genes *Rps18* and *Rplp1* mRNAs (Figure 6(e-g)), while the normal feeding group of *nsp1* transgenic mice did not display significant differences from WT mice. These results indicate that MERS-CoV nsp1 might affect the body weight growth of mice by impairing mitochondrial and ribosomal functions.

## Discussion

After viral infection, a large number of viral components, including viral proteins and nucleic acids are produced. Along with this process, a series of interactions will occur between the virus and host cells, such as the antiviral response of the host cells and viral proteins antagonizing the immune response of the host cells. Highly pathogenic coronaviruses such as SARS-CoV, MERS-CoV, and SARS-CoV-2 can cause severe multiple organ failure in humans, which is associated with immune system disorders, oxidative stress [5–7] and cellular metabolic dysfunction [8–12]. However, the key proteins of SARS-CoV, MERS-CoV, and SARS-CoV-2 that directly damage cellular metabolic processes have not been fully clarified. Sims AC, et al showed the mRNAs of ribosomal protein genes and oxidative phosphorylation genes were downregulated in MERS-CoV infected human lung microvascular endothelial cells [34], but the major constituent of MERS-CoV was unclear. Our present study demonstrated that MERS-CoV nsp1 could damage cellular metabolic processes by specifically downregulating the mRNAs of the ribosomal protein genes and the oxidative phosphorylation protein genes in host cells, resulting in a decrease in cell viability and disruption of metabolism in tissues and organs.

Nsp1 derived from SARS-CoV, MERS-CoV, and SARS-CoV-2 is an endonuclease. Previous studies have demonstrated that the substrate of SARS-CoV nsp1 is mainly the mRNA of the host cells, and the cleavage is the 5’ untranslated region of capped mRNA templates. Although the RNA genome of SARS-CoV also contains a 5’ cap and a 3’ poly A tail structure similar to the host cell mRNA, the leader sequence at the 5’-end can protect the SARS-CoV mRNA from nsp1-induced endonucleolytic RNA cleavage [17]. MERS-CoV nsp1 has been shown to selectively degrade mRNAs from nuclear transcription but spare viral mRNAs of cytoplasmic origin [16]. Here, our data showed that MERS-CoV nsp1 exhibited similar functions to SARS-CoV-2 nsp1, specifically downregulating the mRNA cluster associated with translation, mitochondrial function, and antigen presentation in host cells [35].

Generally, mRNAs in cells combine with various RNA-binding proteins to form membrane-less organelles by liquid-liquid phase separation, such as P-bodies and SGs, which are also referred to as cytosolic biomolecular condensates. The regionalization of proteins and mRNAs in biomolecular condensates can make biological processes more efficient. SARS-CoV nsp1 is localized in the cytoplasm but not in the nucleus [15,36], while SARS-CoV-2 nsp1 is located in both the cytoplasm and nucleus in the form of granules [37]. Here, we found that MERS-CoV nsp1 also aggregated into granules similar to SARS-CoV-2 nsp1. Therefore, it is speculated that MERS-CoV nsp1 may have similar functions as SARS-CoV-2 nsp1, but is different from SARS-CoV nsp1. Indeed, MERS-CoV nsp1 shares many similarities with SARS-CoV-2 nsp1, such as altering the host gene expression level and locating in some granules. However, there are also some differences between them. SARS-CoV and SARS-CoV-2 nsp1 were reported to suppress protein translation by interacting with the 40S ribosomal subunit. However, MERS-CoV nsp1 did not bind stably to the 40S subunit [16,20,21], indicating it displayed different biological functions from SARS-CoV-2 nsp1. The precise mechanism by which MERS-CoV nsp1 inhibits protein translation needs to be further explored.

Cellular mRNAs are dynamically distributed in the cytoplasm, P bodies, and SGs [22,38], and diverse ribonucleoprotein complexes control the processing, degradation, and translation of these mRNAs [39]. Arnaud et al. sorted P-bodies using further fluorescence-activated particle sorting (FAPS). The RNA sequencing results showed that pre-sorted fractions contained more transcripts of ribosomal protein genes, oxidative phosphorylation protein genes, and antigen presentation genes than sorted P-bodies [32]. In addition, Anthony et al. demonstrated that pre-sorted fractions contain more transcripts of ribosomal protein genes and oxidative phosphorylation protein genes than sorted SGs [33]. These results suggest that certain ribonucleoprotein complexes in the pre-sorted fraction contain mRNAs of ribosomal protein genes and oxidative phosphorylation protein genes. In addition to P-bodies and SGs, we found that lysosomes, mitochondria, ribosomes, and nsp1-located granules were also present in the pre-sorted fraction. Interestingly, IP-MS analysis demonstrated that MERS-CoV nsp1-located granules are intracellular non-membrane-bound organelles containing a large number of RNA-binding proteins. Importantly, in addition to the translation process, the proteins in nsp1-located granules are mainly related to ribonucleoprotein complex biogenesis and mRNA metabolic process; therefore, nsp1-located granules are a ribonucleoprotein complex similar to P-bodies and SGs in the cells. Furthermore, RIP analysis demonstrated that the nsp1-located granules contained a large number of mRNAs of ribosomal protein genes and oxidative phosphorylation protein genes. These findings suggest that MERS-CoV nsp1 selectively downregulates the mRNAs of ribosomal protein genes and oxidative phosphorylation protein genes by localizing in novel ribonucleoprotein complexes that are especially rich in these mRNAs.

Several publications by Shinji Makino and colleagues showed C-terminal V5-tagged MERS-CoV nsp1 diffused spread throughout the nucleus and cytoplasm in Vero E6 cells instead of granule formation [16]. We think the main reasons might be that the V5-tag affects the localization of nsp1 in cells. In our study, we observed nsp1 located in some granules in HEK293T cells transfected with GFP-tag MERS-CoV nsp1. In order to exclude the influence of the tag protein, we further detected directly the wild type nsp1 by using the nsp1 antibody, which confirm MERS-CoV nsp1 located in novel granules. Similar phenomena were also observed in human A549 cells (data not shown). In addition, Shinji Makino and colleagues detected nsp1 in Vero E6 cells while we used HEK293T cells and A549 cells, whether MERS-CoV nsp1 has a different location in different cells still needs further study.

The above-mentioned effects of MERS-CoV nsp1 in cells were also observed in nsp1 transgenic mice. MERS-CoV nsp1 expression slowed body weight gain significantly, but did not induce death. However, when the mice were injected with poly(I:C) plus D-galactosamine, MERS-CoV nsp1 overexpression significantly enhanced the sensitivity of the mice to poly(I:C) plus D-galactosamine-induced death. At the same time, the mRNA levels of ribosomal protein genes and oxidative phosphorylation protein genes in the lungs of nsp1 transgenic mice decreased. This suggests that the metabolic process in MERS-CoV nsp1 transgenic mice was inhibited, reducing the responsiveness to external stimuli and rendering the cells more prone to damage. Apart from the lungs, MERS-CoV nsp1 may also cause a decrease in the mRNA levels of ribosomal protein genes and oxidative phosphorylation protein genes in other organs. This may aggravate organ failure induced by viral infection. Therefore, MERS-CoV nsp1 is an important virulence protein that promotes mouse death under certain conditions.

Our present study verified that MERS-CoV nsp1 could inhibit cellular energy metabolism and protein synthesis processes by selectively downregulating the mRNAs of ribosomal protein genes and oxidative phosphorylation protein genes in HEK293T, A549 cells and lung tissues of mice. Importantly, we found that MERS-CoV nsp1 is located in a novel ribonucleoprotein complex containing a large amount of these mRNAs, restricting the substrate mRNAs of MERS-CoV nsp1. Furthermore, the types of mRNA in the MERS-CoV nsp1-located complex are different from those in P-bodies and SGs. Whether this is because of the differences in the RNA-binding proteins among these granules, and which RNA-binding proteins interact with MERS-CoV nsp1 directly, are unknown. Further research is necessary to identify the MERS-CoV nsp1 target protein.

## Materials and methods

### Mammalian cells

HEK293T and A549 cell lines were purchased from ATCC (Manassas, VA, USA). HEK293T cells were cultured in DMEM with 10% fetal bovine serum (FBS), and A549 cells were cultured in 1640 medium with 10% FBS.

### Mice

MERS-CoV nsp1 transgenic mice were generated by knocking-in the nsp1 CDS fragment into the mouse genome using the transposase TMGI system. All mice were generated on or extensively backcrossed to a C57/BL6 background. The F0 generation MERS-CoV nsp1 transgenic mice were mated with WT mice. The offspring of nsp1 transgenic-positive mice were further mated with WT mice. The F3 generation of MERS-CoV nsp1 transgenic mice was used for experiments. Mice were genotyped by PCR using the following primers: 5’-ATGTCTTTCGTGGCTGGTGTGA-3’ (forward) and 5’- ACCGCCAATCAACTTCTTAAGC-3’ (reverse). All animal experiments and protocols were approved by the Institutional Animal Care and Use Committee of the Shandong University. All operations and experiments were performed according to the international guidelines concerning the care and treatment of experimental animals.

### Reagents and antibodies

By cooperating with KWINBON (Beijing, China), mouse monoclonal anti-MERS-CoV nsp1 antibody was prepared in our laboratory; Rabbit monoclonal anti-LAMP1 antibody (6502, CST); Rabbit polyclonal anti-RPS18 antibody (A11687, Abclonal); Rabbit polyclonal anti-LSM14A antibody (A16682, Abclonal); Rabbit polyclonal anti-G3BP1 antibody (A14836, Abclonal); Rabbit polyclonal anti-COX4I1 antibody (A6564, Abclonal); TRIzol™ Reagent (15,596–026, Invitrogen); RNase inhibitor (R0102, Beyotime); Protease inhibitor (B14001, bimake); Phosphatase inhibitor (B15001, bimake); DNase I (D7073, Beyotime); Proteinase K (TIANGEN, RT403); poly(I:C) (APExBIO, B5551); D-galactosamine (Beyotime, ST1213); Tetracyclin (T8180, Solarbio); Mito-Tracker Red CMXRos (Beyotime, C1071S); Cell Counting Kit-8 (CCK-8) (K1018, APExBIO); Cell Cycle Analysis Kit (CY2001, Tianjin Sungene Biotech Co, Ltd); Cell Mitochondrial Stress Test Kit (103,015–100, Agilent Seahorse); Mitochondrial membrane potential assay kit with JC-1 (C2006, Beyotime).

### MERS-CoV plasmid cloning

The initial MERS-CoV templates were provided by Dr. Zhengli Shi (Wuhan Institute of Virology, Chinese Academy of Sciences) as a gift. We cloned the ORFs into a lentiviral expression vector pcDH and a non-viral expression vector pcDNA3.1. GFP-tagged MERS-CoV nsp1, mCherry-tagged G3BP1, LSM14A, and MERS-CoV nsp1-CD were constructed using standard molecular biology techniques.

### CCK8 assay

HEK293T cells were plated in 96-well plates at 5,000 cells/well. MERS-CoV ORF plasmids (100 ng each) were transfected into HEK293T cells. Then, 36 h after transfection, 10 μL CCK8 was added to each well. The culture plates were incubated at 37°C for 2 h, and the absorbance at 450 nm was determined using a microplate reader (Bio-Rad, Hercules, CA).

### Cell cycle analysis

HEK293T cells were seeded into a 12-well plate at 50,000 cells/well. One-microgram MERS-CoV nsp1, nsp1-CD, and control vector were transfected into HEK293T cells. Cell cycle analysis was performed 36 h after transfection, as described previously [40].

### Wound healing

HEK293T cells were seeded into 12-well plates. After 12 h h, the cells were scratched in a straight line using a sterile pipette tip. After washing with PBS, photographs of the scratch wound were recorded at 0 and 24 h.

### RNA sequencing

HEK293T cells were seeded into a 6-well plate, and 12 h later, 1 μg MERS-CoV nsp1, nsp1-CD, and control vector were transfected. These cells were collected 36 h post-transfection, and total mRNA was extracted using TRIzol. cDNA libraries and RNA sequencing as described in a previous report [32].

### Real-time PCR quantification

Total RNA was extracted using TRIzol reagent, and mRNA expression was detected by real-time PCR (qPCR).

### Immunoblot analysis

HEK293T cells or organelles were lysed in SDS lysis buffer (50 mM Tris-HCl pH 6.8, 4% SDS, 0.01% bromophenol blue, 20% glycerol, 100 mM DTT). The lysates were incubated at 97°C for 15 min and analyzed using standard immunoblot procedures. The following antibodies were used: RPS18, RPLP1, COX4I1, COX5B, nsp1, C1QBP, G3BP1, LSM14A, and LAMP1.

### Electron microscopic examination

HEK293T cells were transfected with pcDNA3.1, pcDNA3.1-nsp1, and pcDNA3.1-nsp1-CD overexpression vectors for 36 h, collected, and washed twice with pre-cooled PBS. The subsequent procedures were performed as described previously [41].

### Confocal microscopy

A549 cells transfected with MERS-CoV nsp1, nsp1-CD, or empty vector were incubated with MitoTracker Red for 30 min. The cells were then fixed with 4% paraformaldehyde at 37°C for 15 min and observed under a confocal microscope. For the localization of nsp1 in cells, GFP-tagged nsp1 and mCherry-tagged G3BP1 and LSM14A were co-transfected into HEK293T cells, or stained with MitoTracker Red CMXRos. Then, the cells were fixed with 4% paraformaldehyde at 37°C for 15 min and observed under a confocal microscope. Nuclei were stained with DAPI.

### Cell mitochondrial stress test

A549 or HEK293T cells transfected with MERS-CoV nsp1, nsp1-CD, or empty vector were plated into the seahorse mitochondrial stress test cell plate at 5000 cells/well. Twelve hours later, the cell mitochondrial stress test was performed using the Cell Mitochondrial Stress Test Kit.

### JC-1 assay

HEK293T cells were plated in a 12-well plate at 50,000 cells/well. One-microgram of MERS-CoV nsp1, nsp1-CD, and control vector were transfected into HEK293T cells. After 36 h, JC-1 staining was performed using the mitochondrial membrane potential assay kit and detected by flow cytometry.

### Nsp1 granule sorting

HEK293T cells were plated in a 10 cm dish at 30% confluence. Three-micrograms of nsp1-GFP was transfected into HEK293T cells. After 36 h, the cells were collected and washed twice with pre-cooled PBS, suspended in lysis buffer (50 mM Tris, pH 7.4, 1 mM EDTA, 150 mM NaCl, 0.2% Triton X-100) in the presence of 100 U/mL ribonuclease inhibitor, protease inhibitor, and phosphatase inhibitor. To accelerate lysis, extracts were dounced 20 times. The lysate was centrifuged for 5 min at 200 × *g* to separate the nuclei. One part of the supernatant was added to agarose beads coated with GFP antibody and incubated overnight at 4°C. The beads were then washed three times with 1 mL NP40 lysis buffer I (50 mM Tris -HCl pH7.4, 300 mM NaCl, 1 mM MgCl_2_, 0.05% NP40, 2 mM EDTA, 1 mM DTT, 100 U/mL ribonuclease inhibitor and NP40 lysis buffer II(50 mM Tris-HCl pH7.4, 150 mM NaCl, 1 mM MgCl_2_, 0.05% NP40, 2 mM EDTA, 1 mM DTT, 100 U/mL ribonuclease inhibitor. The beads were suspended in PBS. The remaining supernatant was centrifuged at 10,000 × *g* for 7 min and re-suspended in PBS.

### IP-MS

HEK293T cells were plated in a 10 cm dish at 30% confluence. Three-micrograms of MERS-CoV nsp1 was transfected into HEK293T cells. After 24 h, the cells were collected and washed twice with pre-cooled PBS. Cell pellets were suspended in 1 mL of NP40 lysis buffer (25 mM Tris-HCl pH7.5, 150 mM KCl, 2 mM EDTA, 0.5% NP40, 1 mM NaF, 1 mM DTT, ribonuclease inhibitor, protease inhibitor and phosphatase inhibitor. To accelerate lysis, extracts were dounced 20 times. The lysate was centrifuged for 5 min at 200 × *g* to remove the precipitate. The supernatant was divided into two parts. IgG and nsp1 antibodies with magnetic beads were added to the supernatant and incubated overnight at 4°C. The beads were then washed three times with 1 mL NP40 lysis buffer I (50 mM Tris-HCl pH7.4, 300 mM NaCl, 1 mM MgCl_2_, 0.05% NP40, 2 mM EDTA, 1 mM DTT, 100 U/mL ribonuclease inhibitor and NP40 lysis buffer II (50 mM Tris-HCl pH7.4, 150 mM NaCl, 1 mM MgCl_2_, 0.05% NP40, 2 mM EDTA, 1 mM DTT, 100 U/mL ribonuclease inhibitor. The beads were then lysed in SDS lysis buffer (50 mM Tris-HCl pH 6.8, 4% SDS, 0.01% bromophenol blue, 20% glycerol, 100 mM DTT) and incubated for 15 min at 97°C. After SDS-PAGE gel separation and Coomassie brilliant blue staining, protein strips were cut out for mass spectrometry.

### RIP

HEK293T cells were plated in a 10 cm dish at 30% confluence. Three-micrograms of MERS-CoV nsp1 was transfected into HEK293T cells. Then, 24 h after cell transfection, the cells were collected and washed twice with pre-cooled PBS. Cell pellets were suspended in lysis solution I (50 mM Tris, pH 7.4, 1 mM EDTA, 150 mM NaCl, 0.2% Triton X-100), 100 U/mL ribonuclease inhibitor, and protease inhibitor. Phosphatase inhibitor was added and the extracts were dounced 20 times. The lysate was centrifuged for 5 min at 200 × *g* to remove the nuclei. Supernatants were further spun for 7 min at 10,000 × *g*, and pellets were resuspended in 1 mL of NP40 lysis buffer (25 mM Tris-HCl pH7.5, 150 mM KCl, 2 mM EDTA, 0.5% NP40, 1 mM NaF, 1 mM DTT, 100 U/mL ribonuclease inhibitor, protease inhibitor, phosphatase inhibitor. The lysate was incubated with magnetic beads and the nsp1 antibody overnight at 4°C. The beads were then washed three times with 1 mL NP40 lysis buffer I (50 mM Tris -HCl pH7.4, 300 mM NaCl, 1 mM MgCl2, 0.05% NP40, 2 mM EDTA, 1 mM DTT, 100 U/mL ribonuclease inhibitor and NP40 lysis buffer II(50 mM Tris-HCl pH7.4, 150 mM NaCl, 1 mM MgCl_2_, 0.05% NP40, 2 mM EDTA, 1 mM DTT, 100 U/mL ribonuclease inhibitor. The beads were suspended in PBS. To eliminate the remaining DNA and protein, DNase I was added for 30 min at 10°C, and proteinase K was added for 30 min at 65°C. RNA was extracted using TRIzol reagent and detected by real-time PCR.

### Poly(I:C)/D-galactosamine-induced murine death

Four to five-week-old sex-and age-matched WT mice (purchased from the Beijing HFK Bioscience) and MERS-CoV nsp1 transgenic mice were fed with either a standard diet or a tetracycline diet (2 mg/mL tetracycline) for 2 weeks. During this period, the body weight of the mice was measured daily. After 2 weeks, the mice were injected intraperitoneally with poly(I:C) (2 μg/g body weight) plus D-galactosamine (0.25 mg/g body weight). The survival of the injected mice was monitored every 12 h.

## Supplementary Material

Supplemental Material

## Data Availability

The authors confirm that the data supporting the findings of this study are available within the article and its supplementary materials

## Statistical analysis

Statistical significance was calculated using an unpaired Student’s t test (two-tailed), **p* < 0.05, ***p* < 0.01, ****p* < 0.001, *****p* < 0.001. Results are shown as arithmetic means ± SD. GraphPad Prism 6 was used for statistical analysis.
