# Supplementary figures and images for: MERS-CoV nsp1 impairs the cellular metabolic processes by selectively downregulating mRNAs in a novel granules
Source: Virulence. 2022 Feb 6;13(1):355–69. doi: 10.1080/21505594.2022.2032928 (PMC8824216; doi:10.1080/21505594.2022.2032928)

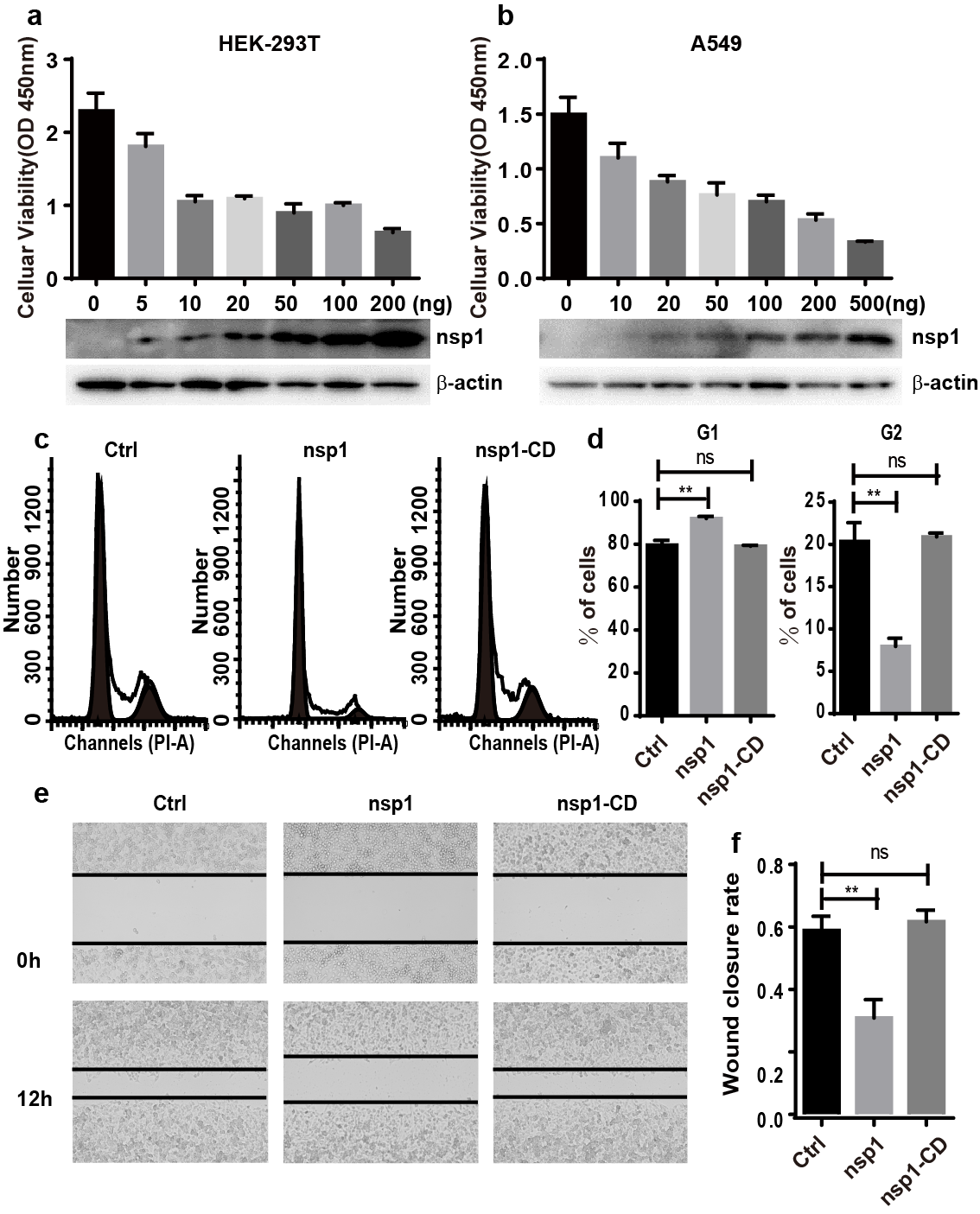

Supplement: Supplemental Material [file KVIR_A_2032928_SM1993.zip › supplementary/Fig.S1.tif]

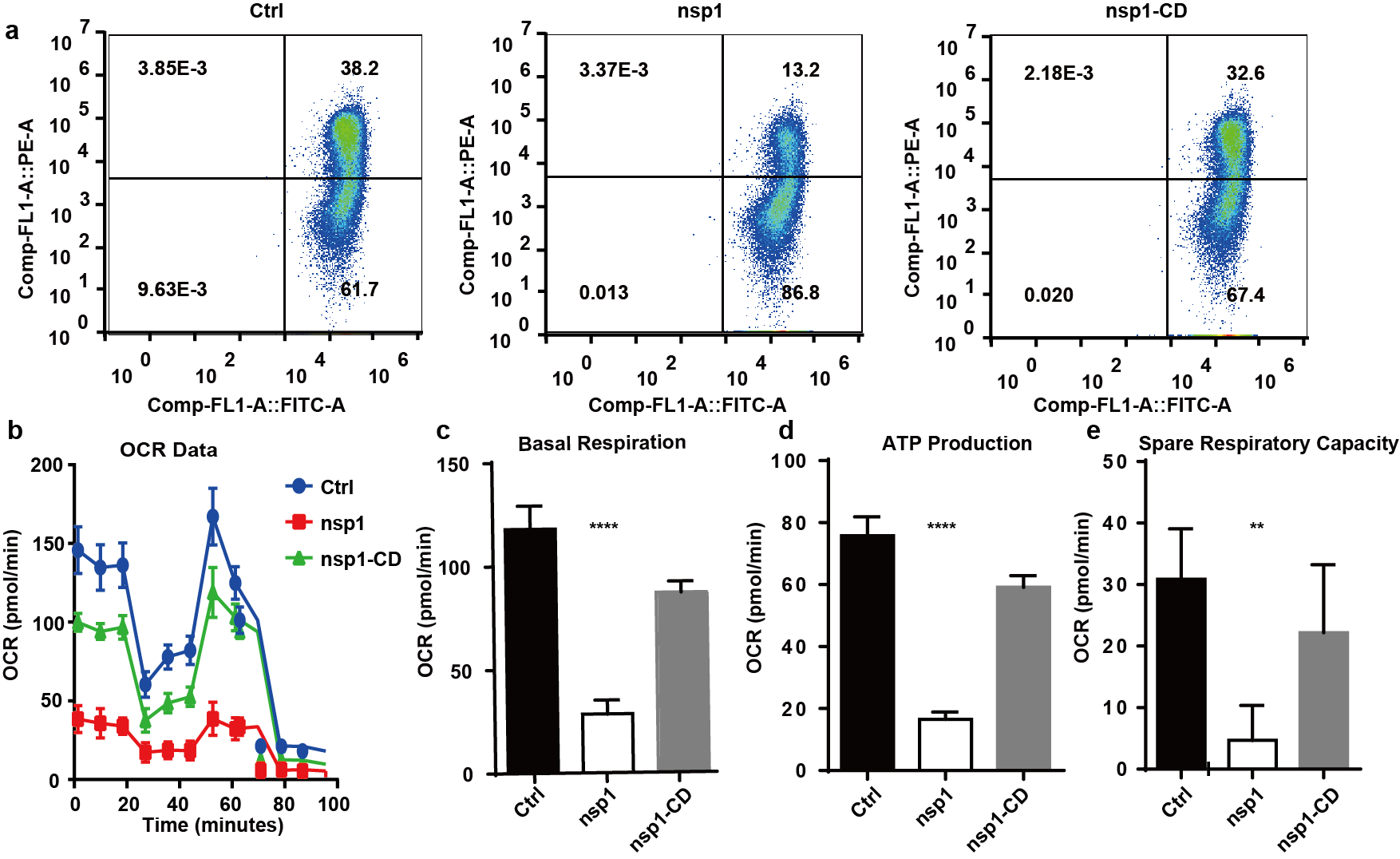

Supplement: Supplemental Material [file KVIR_A_2032928_SM1993.zip › supplementary/Fig.S2.tif]

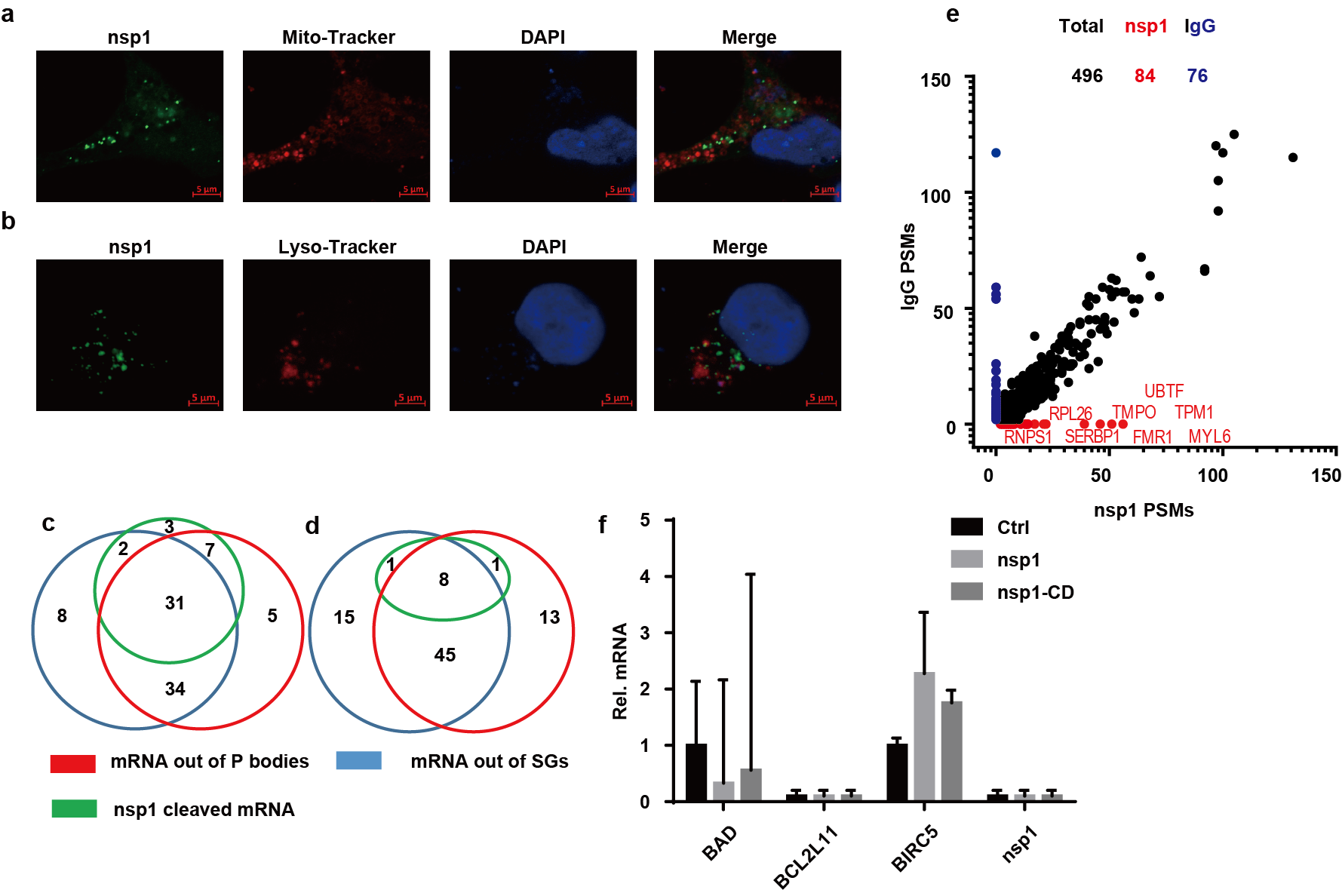

Supplement: Supplemental Material [file KVIR_A_2032928_SM1993.zip › supplementary/Fig.S3.tif]
